# Targeting RAS‐converting enzyme 1 overcomes senescence and improves progeria‐like phenotypes of ZMPSTE24 deficiency

**DOI:** 10.1111/acel.13200

**Published:** 2020-07-24

**Authors:** Haidong Yao, Xue Chen, Muhammad Kashif, Ting Wang, Mohamed X. Ibrahim, Elin Tüksammel, Gwladys Revêchon, Maria Eriksson, Clotilde Wiel, Martin O. Bergo

**Affiliations:** ^1^ Department of Biosciences and Nutrition Karolinska Institutet Huddinge Sweden; ^2^ Department of Plastic and Cosmetic Surgery Tongji Hospital Tongji Medical College Huazhong University of Science and Technology Wuhan China

**Keywords:** mouse models, prelamin A, progeria, RCE1, ZMPSTE24

## Abstract

Several progeroid disorders are caused by deficiency in the endoprotease ZMPSTE24 which leads to accumulation of prelamin A at the nuclear envelope. ZMPSTE24 cleaves prelamin A twice: at the third carboxyl‐terminal amino acid following farnesylation of a *–CSIM* motif; and 15 residues upstream to produce mature lamin A. The carboxyl‐terminal cleavage can also be performed by RAS‐converting enzyme 1 (RCE1) but little is known about the importance of this cleavage for the ability of prelamin A to cause disease. Here, we found that knockout of *RCE1* delayed senescence and increased proliferation of *ZMPSTE24*‐deficient fibroblasts from a patient with non‐classical Hutchinson‐Gilford progeria syndrome (HGPS), but did not influence proliferation of classical *LMNA*‐mutant HGPS cells. Knockout of *Rce1* in *Zmpste24*‐deficient mice at postnatal week 4–5 increased body weight and doubled the median survival time. The absence of *Rce1* in *Zmpste24*‐deficient fibroblasts did not influence nuclear shape but reduced an interaction between prelamin A and AKT which activated AKT‐mTOR signaling and was required for the increased proliferation. Prelamin A levels increased in *Rce1*‐deficient cells due to a slower turnover rate but its localization at the nuclear rim was unaffected. These results strengthen the idea that the presence of misshapen nuclei does not prevent phenotype improvement and suggest that targeting RCE1 might be useful for treating the rare progeroid disorders associated with *ZMPSTE24* deficiency.

## Graphical abstract

A deficiency in the protease ZMPSTE24 causes rare forms of accelerated aging. Here, we show that knockout of a related protease, RCE1, prevents carboxyl‐terminal processing of their shared substrate prelamin A, which disrupts an interaction with AKT and thereby stimulates AKT‐mTOR signaling. Knockout of RCE1 thus overcame senescence of cultured *ZMPSTE24*‐deficient cells and improved survival of *Zmpste24*‐deficient mice.

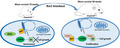

Hutchinson‐Gilford progeria syndrome (HGPS) is typically caused by *LMNA* mutations that lead to accumulation at the nuclear rim of a shortened form of prelamin A called progerin (Eriksson et al., 2003; De Sandre‐Giovannoli et al., 2003). However, atypical HGPS can be caused by mutations in the endoprotease ZMPSTE24 which lead to accumulation of full‐length prelamin A (Barrowman and Michaelis, 2009). *ZMPSTE24* mutations also underlie mandibuloacral dysplasia (MAD) and restrictive dermopathy (RD), which is a mild progeroid disorder, and a severe developmental disorder, respectively (Barrowman and Michaelis, 2009; Michaelis and Hrycyna, 2013).

Prelamin A undergoes four modifications at a carboxyl‐terminal *CSIM* motif (Figure S1): farnesylation of the cysteine by farnesyltransferase (FTase); cleavage of the –*SIM* residues by either ZMPSTE24 or RAS‐converting enzyme 1 (RCE1); methylation of the cysteine by isoprenylcysteine carboxyl methyltransferase (ICMT); and removal of the last 15 amino acids by ZMPSTE24 (Barrowman, Hamblet, George, & Michaelis, 2008; Young, Fong, & Michaelis, 2005). Farnesylation and methylation are necessary for progerin's and prelamin A’s ability to cause progeria. Indeed, FTase inhibitors (FTIs) improve nuclear shape abnormalities of *LMNA*‐ and *Zmpste24*‐mutant cells and clinical phenotypes in HGPS patients (Gordon et al., 2018; Young et al., 2005). Targeting ICMT—explored only preclinically—does not affect nuclear shape but overcomes senescence and eliminates bone fractures and increases life span of *Zmpste24*‐deficient mice (Ibrahim et al., 2013).

However, nothing is yet known about the importance of the carboxyl‐terminal –*SIM* cleavage for prelamin A’s ability to cause disease. Because both ZMPSTE24 and RCE1 can catalyze this step, inhibiting it for therapeutic purposes would only be feasible in the setting of *ZMPSTE24* deficiency, where RCE1 activity would be rate limiting. Knockout of *RCE1* might be predicted to have a similar effect as knockout of *ICMT* in the context of *ZMPSTE24* deficiency since they act sequentially and both interventions would prevent methylation (Ibrahim et al., 2013). In this study, we used genetic strategies to address this issue.

We first analyzed cells from a 5‐year‐old male patient with atypical HGPS (PSADFN373) homozygous for an inactivating *ZMPSTE24* mutation (c.1274 T > C). Atypical HGPS and MAD‐B patients exhibit several clinical phenotypes including stunted growth, lipodystrophy, micrognathia, and hair loss, which overlap substantially, albeit not completely, with those of *Zmpste24*‐deficient mice (Bergo et al., 2002; Ibrahim et al., 2013). As expected from the loss of ZMPSTE24, the PSADFN373 cells expressed prelamin A and lamin C but no lamin A (Figure 1a). When *RCE1* expression in these cells was knocked out with CRISPR/CAS9, their proliferation increased (Figure 1b–d). However, *RCE1* knockout in progerin‐expressing cells (classical *LMNA*‐mutant HGPS) did not increase proliferation, likely because ZMPSTE24 can perform the –*SIM*‐cleavage in these cells (Figure S2a–c). Encouraged by these results, we used gene targeting in mice for further studies.

**Figure 1:**
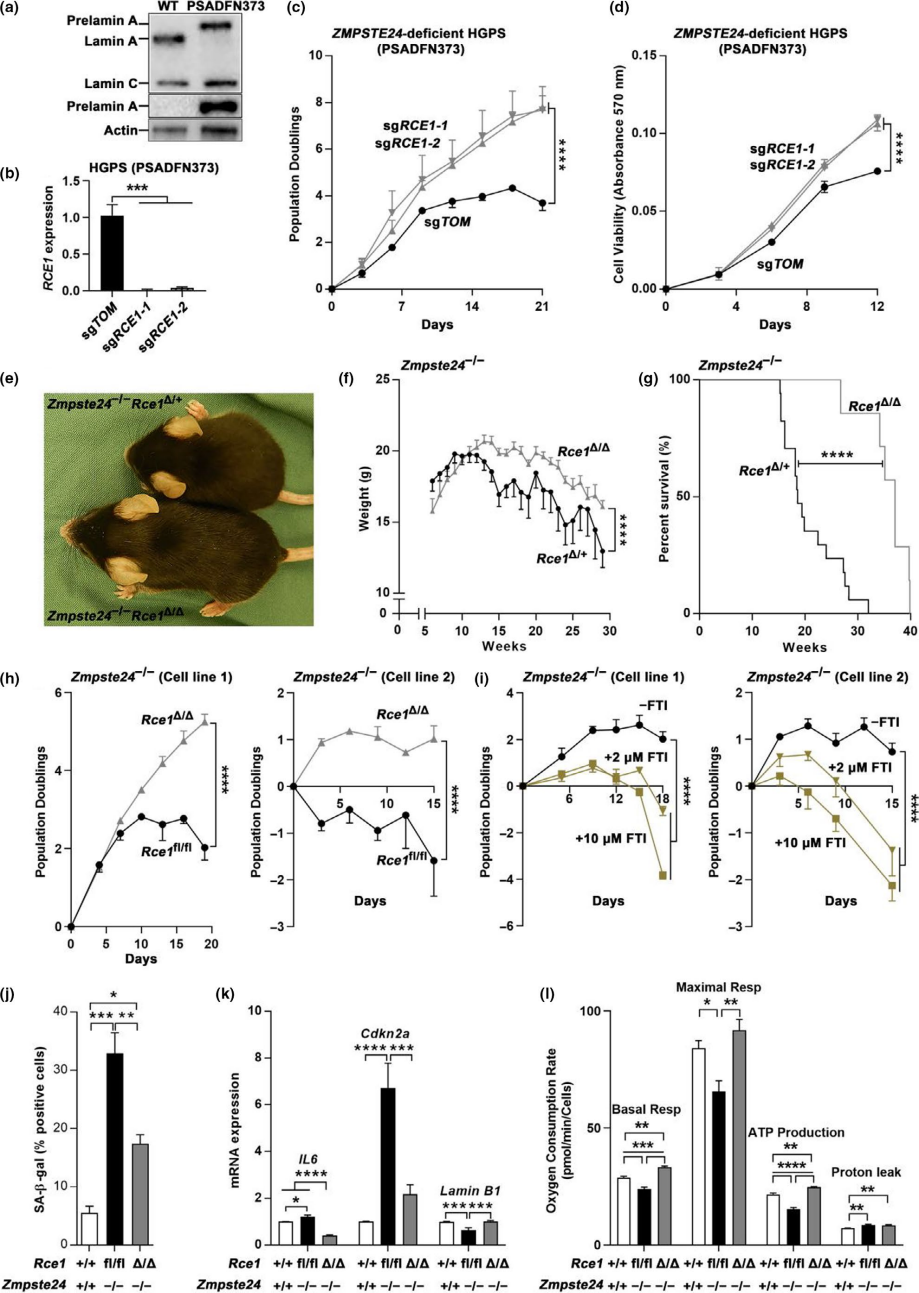
Targeting RCE1 prevents premature senescence in *ZMPSTE24* ^−/−^ fibroblasts and improves survival of *Zmpste24* ^−/−^ mice. (a) Western blots showing accumulation of prelamin A in fibroblasts from a *ZMPSTE24*‐deficient patient (cell line PSADFN373) using lamin A/C antibodies (recognizing the amino terminus of prelamin A, lamin A, and lamin C) and prelamin A antibodies (recognizing the carboxyl terminus); Actin was the loading control. (b) TaqMan analyses showing *RCE1* mRNA levels in the *ZMPSTE24*‐deficient fibroblasts following CRISPR/CAS9‐mediated knockout of *RCE1* with two different sgRNAs; control cells were incubated with nonsense sgRNAs targeting dTomato (sgTOM). (c) Growth curves from population doubling assays of cells from panel b. Data are mean of three technical replicates per cell clone; cells were passage 30. (d) Growth curves from presto blue‐based cell viability assays. Data are mean of six replicates per clone; cells were passage 34. (e) Photograph of 22‐week‐old littermate male mice. (f) Body‐weight curves of male *Zmpste24* mice (*n* = 10) and *Zmpste24* ^−/−^ *Rce1* ^Δ/Δ^ (*n* = 5) mice. (g) Kaplan–Meier plot showing survival of *Zmpste24* ^−/−^ *Rce1* ^Δ/+^ (*n* = 17) and *Zmpste24* ^−/−^ *Rce1* ^Δ/Δ^ (*n* = 7) mice. (h) Growth curves from population doubling assays of primary fibroblasts isolated from two *Zmpste24* ^−/−^ *Rce1* ^fl/fl^ embryos (Cell line 1 and 2); *Cre*‐adenovirus was used to produce *Zmpste24* ^−/−^ *Rce1* ^Δ/Δ^ (i.e., *Rce1* knockout) cells from each parental *Zmpste24* ^−/−^ *Rce1* ^fl/fl^ cell line; β*gal*‐adenovirus was used as control. Data are mean of three replicates per cell line; cells were passage 4. (i) Growth curves from population doubling assays of primary fibroblasts isolated from two *Zmpste24* ^−/−^ *Rce1* ^+/+^ embryos incubated with 2 and 10 µM FTI. Data are mean of three technical replicates per condition; cells were passage 5. (j) SA‐β‐Gal staining assay. Data are mean of three independent cell lines (*n* = 3) assayed in triplicate; cells were passage 8. (k) Expression of senescence markers *IL6*, *Cdkn2a*, *Lamin B1* determined by TaqMan; *β*‐*tubulin* were used as the reference. Data are mean of three cell lines (*n* = 3) assayed in triplicate; cells were passage 9. (l) Seahorse analyses of basal and maximal respiration (Resp.), ATP production, and proton leak. Data are mean of three cell lines assayed in triplicate; cells were in passage 8. * *p* < 0.05; ** *p* < 0.01; *** *p* < 0.005; **** *p* < 0.001

*Rce1* expression in livers of tamoxifen‐injected *Zmpste24* ^–/–^ *Rce1* ^fl/fl^ *Rosa26Cre* ^ERT^ mice (designated *Zmpste24* ^−/−^ *Rce1* ^∆/∆^) was ~65% lower than in livers of *Zmpste24* ^−/−^ *Rce1* ^∆/∆^ controls (Figure S2d,e). Increased body weight and prolonged survival accompanied the reduced *Rce1* expression (38 vs. 19 weeks), which are similar to effects observed with *Icmt* deficiency (Figure 1e–g) (Ibrahim et al., 2013). Because both ‐*SIM*‐cleaved unmethylated prelamin A (i.e., in *Icmt* deficiency) and non‐*SIM*‐cleaved unmethylated prelamin A (i.e., in *Rce1* deficiency) appear to be less toxic than methylated prelamin A, these results suggest that the methyl group contributes to prelamin A’s toxic effect. In contrast to *Icmt* deficiency, *Rce1* knockout did not affect grip strength and bone fractures (Figure S2f,g).

We isolated *Zmpste24* ^–/–^ *Rce1* ^fl/fl^ embryonic fibroblasts and knocked out *Rce1* completely with *Cre*‐adenovirus (Figure S2h). Similar to the results with human cells (i.e., Figure 1c,d), *Rce1* knockout increased proliferation of *Zmpste24* ^–/–^ cells (Figure 1h and Supporting Information Figure S2i), but had no impact on *Zmpste24* ^+/+^ cells (Figure S2j). An FTI dose‐dependently reduced proliferation of *Zmpste24* ^–/–^ cells and prevented the increase in proliferation induced by the *Rce1* knockout (Figure 1i and Figure S2k). These results confirm earlier findings that *Rce1* deficiency is compatible with cell proliferation whereas FTase inhibition—and knockout of the FTase β subunit—reduces or blocks it (Lee et al., 2010; Liu et al., 2010; Wahlstrom et al., 2007).

Consistent with increased proliferation, *Rce1* knockout reduced senescence‐associated β‐galactosidase activity of *Zmpste24* ^–/–^ cells, and the expression of senescence markers *Il6* and *Cdkn2a*; and increased *Lmnb1* expression (Figure 1j,k). In line with earlier studies in HGPS cells (Rivera‐Torres et al., 2013), basal and maximal respiration and ATP production were lower in *Zmpste24* ^–/–^ than wild‐type cells. Knockout of *Rce1* increased oxygen consumption rates and normalized those metabolic parameters; they were even increased slightly but significantly above baseline (Figure 1l).

Misshapen nuclei are a hallmark of progerin‐ and prelamin A‐expressing cells in culture and FTIs improve this phenotype (Capell et al., 2005; Toth et al., 2005). *Rce1* knockout, however, did not influence nuclear shape of *Zmpste24* ^–/–^ cells (Figure 2a). Consistent with previous studies (Ibrahim et al., 2013), AKT‐mTOR signaling was low in *Zmpste24* ^–/–^ cells as judged by Western blots of phospho‐AKT and phospho‐S6 (Figure 2b). Knockout of *Rce1* restored phospho‐AKT and phospho‐S6 levels, and disrupted the prelamin A–AKT interaction (Figure 2b,c). Moreover, an AKT inhibitor prevented the proliferation increase induced by *Rce1* knockout (Figure 2d); and an AKT activator increased proliferation of naïve *Zmpste24* ^–/–^ cells (Figure 2e). These data suggest that prelamin A in *Zmpste24*‐deficient cells binds AKT and prevents its phosphorylation and signaling; when the last three amino acids of prelamin A are retained, as in *Rce1*‐knockout cells, the prelamin A–AKT interaction is disrupted and subsequent AKT activation drives increased proliferation.

**Figure 2:**
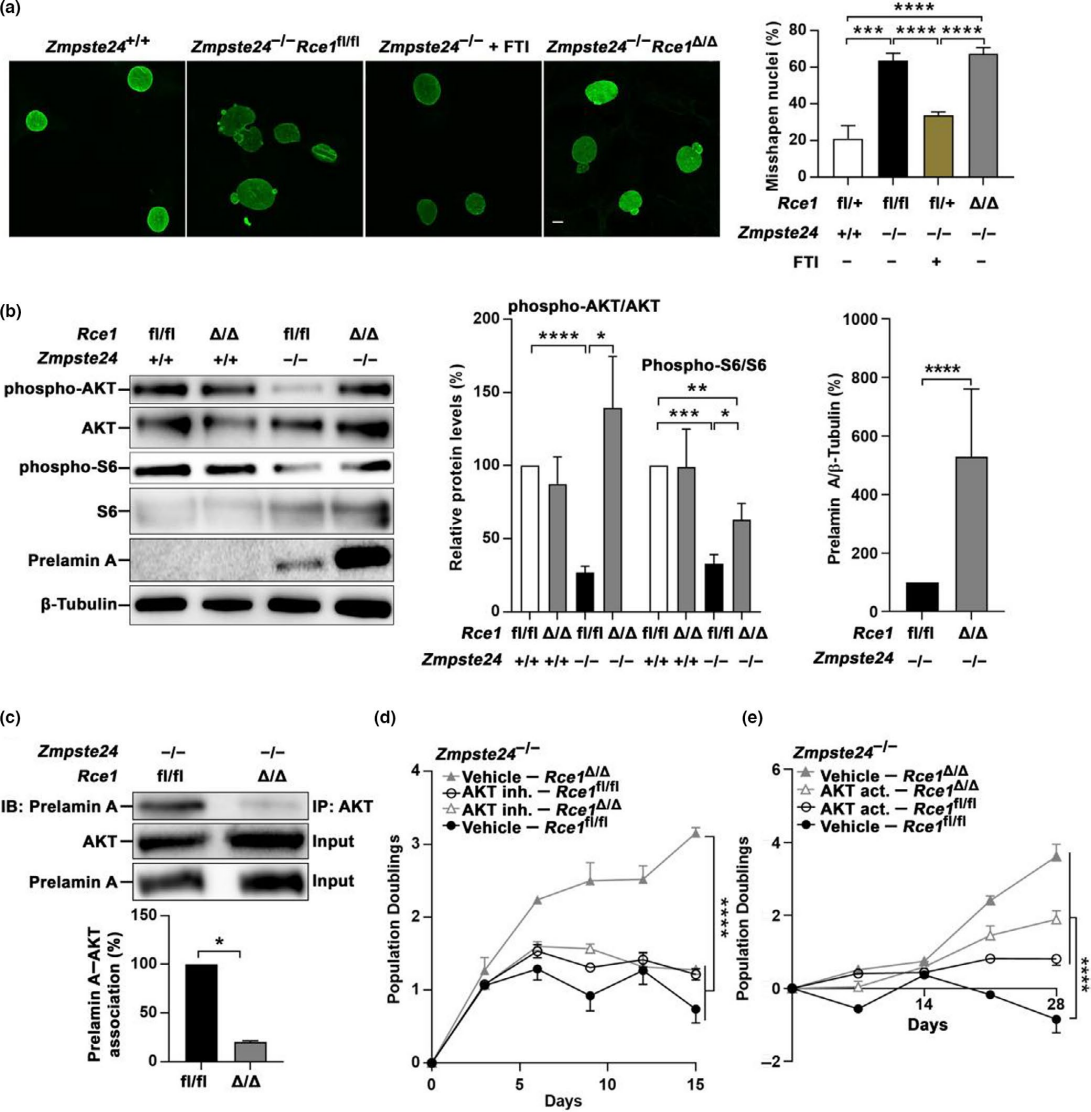
*Rce1* knockout prevents premature senescence of *Zmpste24* ^−/−^ fibroblasts by increasing AKT pathway signaling, but has no impact on nuclear shape. (a). Left, confocal images of representative nuclei in primary mouse embryonic fibroblasts stained with LAP2β antibodies; FTI (FTI‐276) concentration was 2 µM. Right, quantification of misshapen nuclei. Data are mean of three cell lines (*n* = 3) per condition; cells were passage 8. (b) Left, Western blots of fibroblast lysates showing steady‐state levels of phosphorylated and total AKT and S6, and prelamin A; β‐tubulin was the loading control. Middle and right, ratio of phosphorylated and total AKT and S6 (middle) and ratio of prelamin A and β‐tubulin (right) determined by densitometry of protein bands. Data are mean of three cell lines (*n* = 3) per genotype; cells were passage 8. (c) Upper, immunoprecipitation (IP) and Western blot (WB) showing an *Rce1*‐dependent interaction between prelamin A and AKT in *Zmpste24* ^–/–^ fibroblasts. The lysates were also used directly for Western blot of total AKT and prelamin A levels (input). Lower, prelamin A–AKT interaction determined by densitometry of protein bands. Data are mean of three cell lines (*n* = 3) per genotype and normalized first to total AKT and then to control (*Zmpste24* ^–/–^ *Rce1* ^fl/fl^); cells were passage 8. (d, e) Growth curves from population doubling assays of fibroblasts incubated with an AKT inhibitor (20 µM, GSK690693) (d) and an AKT activator (5 µM, SC‐79) (e). Data are mean of triplicate analyses per condition; similar results were obtained with two cell lines each analyzed in two experiments; cells were passage 4. Scale bar, 20 µm, * *p* < 0.05; ** *p* < 0.01; *** *p* < 0.005; **** *p* < 0.001

Consistent with absent RCE1 activity, RAS proteins increased in the cytosolic fraction and decreased in the membrane fraction of *Zmpste24* ^–/–^ *Rce1* ^∆/∆^ cells; and RAS and prelamin A exhibited a slight electrophoretic mobility shift (Figure S3a,b). Prelamin A was primarily localized at the nuclear membrane in *Zmpste24* ^–/–^ fibroblasts and hepatocytes, and the localization was unaffected by the knockout of *Rce1* (Figure S3c,d).

Data in Figure 2b and Figure S3d revealed that steady‐state levels of prelamin A were higher in *Zmpste24* ^–/–^ *Rce1* ^∆/∆^ than *Zmpste24* ^–/–^ *Rce1* ^fl/fl^ cells (Figure 2b and Figure S3d). When protein synthesis was stopped with cycloheximide, prelamin A disappeared at a slower rate in *Zmpste24* ^–/–^ *Rce1* ^∆/∆^ than *Rce1* ^fl/fl^ cells (Figure S3e). This result suggests that retention of the –*SIM* amino acids reduces prelamin A turnover and increases steady‐state levels.

Previous studies revealed that active AKT can phosphorylate prelamin A at Ser404 and trigger prelamin A degradation (Bertacchini et al., 2013; Cenni et al., 2008). Therefore, the finding that *Rce1* deficiency was associated with increased AKT activity and reduced prelamin A degradation is surprising. However, this effect was also observed with *Icmt* deficiency. The reason behind the opposing results is unclear but one potential explanation is that the absence of the methyl group or retention of the last three amino acids in our two studies prevents binding to proteins that contribute to prelamin A degradation. Future studies should determine whether AKT‐induced Ser404 phosphorylation influences the stability of endogenous prelamin A and senescence in the setting of *Zmpste24* deficiency; and the impact of knocking out *Rce1* and *Icmt*.

Thus, targeting RCE1‐mediated endoproteolysis increased survival and alleviated some phenotypes of *Zmpste24* deficiency in vivo, but the effect was less than that observed by targeting *Icmt* (Ibrahim et al., 2013). A potential explanation is that *Rce1* was knocked out by ~65% in 4‐ to 5‐week‐old mice whereas *Icmt* was knocked out by ~85% throughout development using a hypomorphic allele (Ibrahim et al., 2013). Thus, it is possible that the effects of the *Rce1* knockout in vivo are underestimated. This argument is strengthened by the finding that the knockout of *Rce1* in vitro—which was near complete (Figure S2h)—showed more robust effects, comparable to *Icmt* deficiency.

Interestingly, the phenotypes of *Zmpste24* deficiency improved following *Rce1* knockout despite increased steady‐state levels of farnesylated prelamin A; unaltered localization at the nuclear rim; and lack of effect on nuclear shape. The reduced toxicity of non‐*SIM*‐cleaved prelamin A could potentially be derived from altered protein–protein interactions, including the reduced interaction with AKT which was associated with increased AKT signaling and required for the increased proliferation. But we cannot rule out the possible involvement of other *CAAX*‐protein substrates of RCE1, aside from prelamin A.

A specific RCE1 inhibitor would be required to determine whether targeting this enzyme pharmacologically could be useful in treating disorders of *ZMPSTE24* deficiency—a strategy that would be relevant for MAD‐B and the extremely rare atypical form of HGPS, but not for RD as it is lethal at birth (Hampton, Dore, & Schmidt, 2018). However, such an inhibitor would not be required to completely inhibit RCE1 because 65% of reduced *Rce1* expression was sufficient to double the median survival of *Zmpste24*‐deficient mice.

## CONFLICT OF INTERESTS

The authors declare that no competing interests exist.

## AUTHOR CONTRIBUTIONS

H.Y. designed the study, performed experiments, interpreted data, made figures, and wrote the manuscript; X.C. performed experiments, interpreted data, and made figures; M.K. designed and performed experiments; T.W. performed experiments; M.X.I. designed experiments; E.T. performed mouse experiments; G.R. performed experiments; M.E. designed experiments and interpreted data; C.W. designed and performed experiments, supervised, and wrote the manuscript; M.O.B. designed the study, provided funding, and wrote the manuscript.

## Supporting information

Figure S1‐S3

Supplementary Material

## Data Availability

The data that support the findings of this study are available from the corresponding author upon reasonable request.
