# Supplementary material for: Targeting RAS‐converting enzyme 1 overcomes senescence and improves progeria‐like phenotypes of ZMPSTE24 deficiency
Source: Aging Cell. 2020 Jul 24;19(8):e13200. doi: 10.1111/acel.13200 (PMC7431821; doi:10.1111/acel.13200)
Supplement: Supplementary file 1 — Figure S1‐S3 [file ACEL-19-e13200-s001.docx]

**Online Supporting Information**

**FIGURE LEGENDS**

**Figure S1. Posttranslational processing of prelamin A**. The *CAAX* motif of prelamin A (–*CSIM*) trigger four sequential posttranslational modifications: 1, farnesylation of the cysteine residue (i.e., the “*C*” in *CSIM*) by protein farnesyltransferase (FTase); 2, endoproteolytic cleavage of the last three amino acids (*–SIM*) by RAS-converting enzyme 1 (RCE1) or zinc metalloproteinase ste24 homologue (ZMPSTE24); 3, carboxyl methylation of the newly exposed farnesylcysteine residue by isoprenylcysteine carboxyl methyltransferase (ICMT); and 4, endoproteolytic release of the 15 carboxyl-terminal amino acids, including the methylated farnesylcysteine residue, by ZMPSTE24. In wild-type cells these modifications result in the production of mature lamin A. In *ZMPSTE24*-deficient cells, full-length farnesylated and methylated prelamin A accumulates at the nuclear rim; in HGPS cells, the ZMPSTE24 cleavage site is missing due to an internal truncation, and a shortened form of farnesylated and methylated prelamin A (progerin) accumulates at the nuclear rim.

**Figure S2. Knockout of *Rce1* prevents premature senescence of *Zmpste24*^−/−^ fibroblasts but has no impact on classical *LMNA*-mutant HGPS cell lines.** (**a**) Western blots showing accumulation of progerin in fibroblasts from two HGPS patients (AG01972, AG11513) using lamin A/C antibodies (recognizing the amino terminus of lamin A, progerin, and lamin C); Tubulin was the loading control. (**b**) TaqMan analyses showing RCE1 mRNA levels in HGPS cells following CRISPR/CAS9-mediated knockout of RCE1; control cells were incubated with nonsense sgRNAs targeting dTomato (dTOM). (**c**) Growth curves from presto blue-based cell viability assays of cells in panel b. Data are mean of triplicates. (**d**) PCR-based genotyping of genomic DNA isolated from livers of mice in Figure 1e–g showing the appearance of the *Rce1* knockout (“Δ”) allele in tamoxifen-injected *Zmpste24*^−/−^*Rce1*^fl/fl^*Rosa26Cre*^ERT^ mice; DNA from *Zmpste24*^−/−^*Rce1*^fl/fl^ fibroblasts incubated with *Cre*- and *βgal*-adenoviruses was used as control. (**e**) TaqMan analyses showing *Rce1* expression in livers of mice from Figure 1. Data are mean of three mice per genotype analyzed in duplicate. (**f**) Upper, ventral view of spinal columns of an 18-week-old *Zmpste24*^−/−^*Rce1*^Δ/+^ and a 35-week-old *Zmpste24*^−/−^*Rce1*^Δ/Δ^ mouse; white spots at costovertebral joints are rib fractures. Bottom, number of rib fractures in *Zmpste24*^−/−^*Rce1*^Δ/+^ mice (*n* = 16) and *Zmpste24*^−/−^*Rce1*^Δ/Δ^ mice (*n* = 7). (**g**) Grip strength of 15-week-old mice (*n* = 7/genotype). (**h**) PCR-based genotyping of genomic DNA isolated from *Zmpste24*^−/−^*Rce1*^fl/fl^ cells following incubation with *Cre*- and *βgal*-adenoviruses showing conversion of the floxed allele into the *Rce1* knockout Δ allele in *Cre*-adenovirus-treated cells. (**i**) Growth curves from cell viability assays of primary fibroblasts isolated from two *Zmpste24*^−/−^*Rce1*^fl/fl^ embryos (Cell line 1 and 2, same as in Figure 1h) and incubated with *Cre*- and *βgal*-adenoviruses. Data are mean of six replicates per cell line; cells were passage 4. (**j**) Growth curves from presto blue-based cell viability assays of primary *Zmpste24*^+/+^*Rce1*^fl/fl^ fibroblasts; *Cre*-adenovirus was used to produce *Zmpste24*^+/+^*Rce1*^Δ/Δ^ (i.e., *Rce1* knockout) cells from the parental *Zmpste24*^+/+^*Rce1*^fl/fl^ cell line; *βgal*-adenovirus was used as control. Data are mean of three technical replicates; cells were passage 4. (**k**) Growth curves from population doubling assays of Cell line 1 of panel i; the *Zmpste24*^−/−^*Rce1*^Δ/Δ^ cells were incubated with 2 µM FTI. Data are mean of three replicates per cell line and condition; cells were passage 7. ** *P* < 0.01; *** *P* < 0.005; **** *P* < 0.001.

**Figure S3. *Rce1* deficiency increases prelamin A levels but does not influence nuclear membrane localization.** (**a**) Western blots showing levels of RAS in total lysates (T) and in cytosolic (C) and membrane (M) fractions of cells from Figure 1h; β-Tubulin was the loading control; cells were passage 8. Note slightly reduced electrophoretic mobility of RAS in total lysate (T) of *Zmpste24*^–/–^*Rce1*^Δ/Δ^ cells (right lane, illustrated by thin line) (**b**) Western blots showing levels of prelamin A and lamin C using antibodies recognizing the amino (N) terminus and antibodies recognizing the carboxyl (C) terminus; cells were passage 8. Note slightly reduced electrophoretic mobility of prelamin A in *Zmpste24*^–/–^*Rce1*^Δ/Δ^ cells (right lane, illustrated by thin line). (**c**) Left, immunofluorescence images of nuclei in liver sections stained with prelamin A antibodies (red) and DAPI (blue). Right, prelamin A staining intensity at the nuclear membrane and in the nucleoplasm. Data are mean of livers from three mice per genotype. (**d**) Western blots showing levels of prelamin A in nuclear membrane and nucleoplasm fractions isolated from *βgal*- and *Cre*-adenovirus-infected *Zmpste24*^–/–^*Rce1*^fl/fl^ cells; lamin B2 and nuclear matrix protein p84 were used as loading control in nuclear membrane and nucleoplasm fractions, respectively; cells were passage 8. S, short exposure; L, long exposure. (**e**) Left, western blots showing the disappearance of prelamin A in *Zmpste24*^–/–^*Rce1*^fl/fl^ and *Zmpste24*^–/–^*Rce1*^Δ/Δ^ cells incubated with cycloheximide (CHX, 20 µg/ml) to stop protein synthesis. Right, percent prelamin A remaining over time following cycloheximide administration determined by densitometry of protein bands; each band was normalized to time-point 0 h within each genotype. Data are mean of three cell lines per genotype; cells were passage 8. Scale bar, 20 µm, * *P* < 0.05.

**Experimental procedures**

**Mice**

*Zmpste24*^−/−^ mice (Bergo, Gavino, et al., 2002) were bred with mice harboring conditional *Rce1* knockout alleles (*Rce1*^fl^) (Bergo, Ambroziak, et al., 2002) and a tamoxifen-inducible *Rosa26-Cre* ([Gt(ROSA)26Sortm1(cre/ERT2)Tyj](https://www.jax.org/strain/008463#jump-nav-3)) mice (Ventura et al., 2007) to produce *Zmpste24*^−/−^*Rce1*^fl/fl^*Rosa26Cre*^ERT^ mice, hereafter designated *Zmpste24*^−/−^*Rce1*^fl/fl^. Groups of 4-week-old *Zmpste24*^−/−^*Rce1*^fl/fl^ mice were injected with 100 μl tamoxifen (20 mg/ml in sunflower oil, T5648, Sigma) once daily for five days to generate *Zmpste24*^−/−^*Rce1*^Δ/Δ^ mice, (Δ, delta, or deleted allele). Controls for *Zmpste24* deficiency were littermate *Zmpste24*^−/−^*Rce1*^Δ/+^ and *Zmpste24*^−/−^*Rce1*^+/+^ mice, which were indistinguishable in phenotype and collectively designated *Zmpste24*^−/−^*Rce1*^Δ/+^. Littermate *Zmpste24*^+/^^−^*Rce1*^Δ/+^ and *Zmpste24*^+/−^*Rce1*^Δ/Δ^ mice were used in some experiments as healthy controls. Genotyping was performed by polymerase chain reaction (PCR) on genomic DNA from ear or tail biopsies. Mice were monitored daily and weighed weekly. Grip strength was measured on 15-week-old mice using a Sauter FL Digital Force Gauge (Sauter). Five measurements were recorded per mouse, with > 1 minute rest between each measurement. Mouse experiments were approved by the Animal Research Ethics Committees in Gothenburg and Linköping, Sweden.

**Cell culture and proliferation and senescence assays**

Cell line PSADFN373 homozygous for a *ZMPSTE24* mutation (c.1274T>C) was from the Progeria Research Foundation; cell lines AG01972 and AG11513 heterozygous for the classical HGPS mutation (c.1824C>T) and the control cell line AG03258 from an unaffected parent were from the Coriell Institute. Primary mouse fibroblasts isolated from E13.5–E14.5 embryos were cultured in low-glucose DMEM ([21885025](https://www.thermofisher.com/order/catalog/product/21885025), ThermoFisher) supplemented with 10% fetal bovine serum (FBS, 26140079, ThermoFisher), 1% penicillin/streptomycin (15070063, ThermoFisher), and 1% MEM Non-essential Amino Acid (11140068, ThermoFisher). For adenoviral transduction of mouse fibroblasts, 10^6^ cells were seeded in 100-mm dishes and incubated for 24 h with 10 MOI adenoviruses expressing *Cre* recombinase (Ad5CMV*Cre*) or β-galactosidase (*βgal*; Ad5CMVnt*LacZ*, University of Iowa, Iowa City, IA) in 10 ml cell culture medium. Population doubling proliferation assays were carried out by plating 3 × 10^5^ cells on 100-mm dishes; the cells were trypsinized and counted, and 3 × 10^5^ cells re-seeded every 3 days. AKT inhibitor (20 µM GSK690693; S1113, Sigma-Aldrich), AKT activator (5 µM SC79; 123871, Sigma-Aldrich), and FTase inhibitor (2 and 10 µM FTI-276; F9553, Sigma-Aldrich) were used in some proliferation assays. Cell viability proliferation assays were carried out by plating 1 × 10^3^ cells in 96-well plates; cell viability was determined every three days with PrestoBlue Cell Viability Reagent ([A13262](https://www.thermofisher.com/order/catalog/product/A13262), ThermoFisher); absorbance at 570 and 600 nm was measured with the Multi-mode reader (BioTek). Senescence-associated β-galactosidase (SA‐β-Gal) staining of fibroblasts was performed using the Senescence Detection Kit (9860, Cell Signaling).

**Quantitative PCR**

RNA was isolated with the RNeasy Plus Mini Kit (74136, QIAGEN) and cDNA was synthesized with the iScript cDNA synthesis Kit (170-889, Bio-Rad). Expression of *Il6*, *Cdkn2a*, *Lmnb1*, and *RCE1* was analyzed by reverse transcription quantitative polymerase chain reaction on a CFX384 Real-Time System (Bio-Rad) using Taqman mouse probe sets Mm00446190_m1, [Mm00494449_m1](https://www.thermofisher.com/taqman-gene-expression/product/Mm00494449_m1?CID=&ICID=&subtype=), Mm00521949_m1, and Hs00192923, respectively (ThermoFisher). *β-Tubulin* ([Mm00727586_s1](https://www.thermofisher.com/taqman-gene-expression/product/Mm00727586_s1?CID=&ICID=&subtype=)), *Actin* ([Mm02619580_g1](https://www.thermofisher.com/taqman-gene-expression/product/Mm02619580_g1?CID=&ICID=&subtype=)), *ACTIN* ([Hs99999903_m1](https://www.thermofisher.com/taqman-gene-expression/product/Hs99999903_m1?CID=&ICID=&subtype=)), and *β-TUBULIN* (Hs00801390) (ThermoFisher) were used as reference genes.

**Lentivirus experiments**

Lentiviruses were produced by transfecting 293FT packaging cells (R70007, Life Technologies) with 6 μg lentiviral backbone constructs, 3 μg packaging plasmid psPAX2 (#12260, Addgene), and 1.5 μg envelope plasmid pMD2.G (#12259, Addgene) using the Xfect transfection reagent (631318, Clonetech). Lentiviral supernatants were collected two days after transfection, supplemented with 8 μg/ml polybrene (107689-10G, hexadimethrine bromide; Sigma-Aldrich), and used to transduce target cells; the cells were then incubated for two days with blasticidin (1 μg/ml, A1113903, ThermoFisher). Lentiviral backbones were pLentiCRISPRv2 blast (#98293) expressing a single guide (sg) RNA targeting human *RCE1*. For sgRNA cloning, the lentiCRISPRv2 vector was digested with BsmBI and ligated with BsmBI-compatible pre-annealed oligonucleotides. The following sequences were used: *sgdTomato*, 5'–GGCCACGAGTTCGAGATCGA–3'­; human *sgRCE1_h1*, 5'–GTTCGCTCTTCCAGACGTAG–3'; and human *sgRCE1_h2* 5'–TATGGATTGCCCTTGTGACC–3'.

**Mitochondrial function assay**

Mitochondrial function parameters were measured with the Cell Mito Stress Test kit using the Seahorse XFe96 Analyzer (Agilent) which measures the oxygen consumption rate in real time in live cells. Cells were seeded in Seahorse XF96 microplates (30,000 cells/well) (101085-004, Agilent) and cultured overnight at 37°C in a CO_2_ incubator. Freshly prepared phenol red–free base medium supplemented with HEPES, glucose, pyruvate, and glutamine, and adjusted to pH 7.4 were added to the cells followed by incubation for 45 min at 37°C in a non-CO2 incubator. The assay was carried out at 37°C in the XFe96 Analyzer. Basal and maximal respiration, ATP production, and proton leak were calculated using the Macro excel file provided by the manufacturer. Data were normalized to viable cell numbers obtained from identically-treated additional wells using the Presto Blue Cell Viability assay ([A13262](https://www.thermofisher.com/order/catalog/product/A13262), ThermoFisher).

**Nuclei shape analysis and** **immunofluorescence**

Fibroblasts were cultured on Glass Bottom Microwell Dishes (MatTek) for 24 h, fixed in 4% paraformaldehyde, permeabilized with 0.4% Triton X-100, and blocked with PBS containing 2% bovine serum albumin. The cells were then incubated overnight with antibodies recognizing LAP2β (1:100, 611000, BD Biosciences), followed by incubation for 1 h with secondary antibodies (1:1000, Alexa Fluor Plus 488 goat anti-mouse IgG, A32723, ThermoFisher), and then counterstained for 15 min with DAPI (1:500, 62248, ThermoFisher). For immunofluorescence analyses of mouse liver nuclei, livers where frozen in OCT (Sakura Finetek Japan, Tokyo, Japan) and 5-µm cryosections where fixed in 4% formaldehyde, rinsed with PBS, permeabilized with 100% ice-cold methanol, blocked in 1% BSA, and incubated overnight with antibodies recognizing prelamin A (1:100, MABT345, Millipore). The samples were then rinsed in PBS, incubated for 1 h with secondary antibodies (1:1000, Alexa Fluor 568 goat anti-rat IgG, A11077, Life Technologies), and mounted in Prolong Gold Antifade reagent with DAPI (1:500, 62248, ThermoFisher). Fluorescence images were collected with a Nikon A1R Confocal Microscope (Nikon Instruments, Japan) using 20×/0.75-air and 60×/1.4-oil objectives for LAP2β and prelamin A staining, respectively. Image acquisition settings were: blue channel, 405 nm laser and 450/50 emission filter; green channel, 488 nm laser and 525/50 emission filter; and red channel, 561 nm laser and 595/50 emission filter. The frequency of misshapen nuclei was quantified in LAP2β-stained cells as described (Ibrahim et al., 2013). Nucleoplasm and nuclear membrane staining of prelamin A was quantified by analyzing the fluorescence intensity of ~300 nuclei in three liver sections/mouse.

**Western blot, subcellular fractionation, immunoprecipitation, and protein turnover**

For western blots, fibroblasts were lysed in buffer containing 9 M urea (U0631, Sigma-Aldrich) and protease inhibitor cocktail (78430, ThermoFisher), sonicated and cleared by centrifugation (14,000 × *g* for 10 min). Cytosolic and membrane fractions were isolated with Qproteome Cell Compartment Kit (37502, QIAGEN). Nuclear membrane and nucleoplasm fractions were isolated with Minute Nuclear Envelop Protein Extraction Kit (NE-013) and Minute Detergent-Free Nucleoplasm Isolation Kit (NI-024, Invent Biotechnologies), respectively. Proteins in whole-cell lysates and cellular and nuclear fractions were size-fractionated on 10 or 12% Mini-PROTEAN TGX Stain-Free gels (456-8036, Bio-Rad) and proteins were transferred to nitrocellulose membranes (0.2 μm, 1704158, Bio-Rad). The membranes were incubated with primary antibodies overnight at 4°C and with secondary antibodies for 1 h at room temperature. Primary antibodies were lamin A/C (E-1) (1:100, sc-376248, Santa Cruz), prelamin A (1:500, MABT345, Millipore), lamin B2 (1:100, 33-2100, ThermoFisher), phospho-AKT^Ser473^ (1:1000, 4060), AKT (1:1000, 9272), S6 (1:500, 2317), phospho-S6 (1:1000, 4858, Cell signaling), pan-RAS (1:1000, ab69747, Abcam), nuclear matrix protein p84 (1:500, GTX70220, GeneTex), β-Tubulin (1:1000, T2200, Sigma-Aldrich), and Actin (1:1000, A2228, Sigma-Aldrich). Secondary antibodies were from Jackson Immunoresearch Laboratories. Clarity Western ECL susbstrate (1705061, BioRad) was used for detection of protein bands with the ChemiDoc Touch Imaging system (1708370, Bio-Rad). Immunoprecipitation (IP) was performed with the Dynabeads Protein G Immunoprecipitation Kit (10007D, ThermoFisher). To quantify prelamin A turnover rate, cells were incubated with cycloheximide (20 µg/ml, C4859, Sigma-Aldrich) to stop protein synthesis; lysates were prepared as outlined above.

**Statistics**

Data are mean and standard error of the mean (SEM). We used GraphPad Prism (v.7.0) for statistical analyses; two-way ANOVA was used for cell proliferation curves and degradation assay; log-rank for survival; two-sided *t* test was used when comparing only two groups; and one-way ANOVA for all other variables. Experiments were repeated 2–4 times unless stated otherwise; *n* indicates biological replicates.

**References**

Bergo, M. O., Ambroziak, P., Gregory, C., George, A., Otto, J. C., Kim, E., . . . Young, S. G. (2002). Absence of the CAAX Endoprotease Rce1: Effects on Cell Growth and Transformation. Molecular and Cellular Biology, 22(1), 171-181. doi:10.1128/mcb.22.1.171-181.2002

Bergo, M. O., Gavino, B., Ross, J., Schmidt, W. K., Hong, C., Kendall, L. V., . . . Young, S. G. (2002). Zmpste24 deficiency in mice causes spontaneous bone fractures, muscle weakness, and a prelamin A processing defect. Proc Natl Acad Sci U S A, 99(20), 13049-13054. doi:10.1073/pnas.192460799

Ibrahim, M. X., Sayin, V. I., Akula, M. K., Liu, M., Fong, L. G., Young, S. G., & Bergo, M. O. (2013). Targeting isoprenylcysteine methylation ameliorates disease in a mouse model of progeria. Science, 340(6138), 1330-1333. doi:10.1126/science.1238880

Ventura, A., Kirsch, D. G., McLaughlin, M. E., Tuveson, D. A., Grimm, J., Lintault, L., . . . Jacks, T. (2007). Restoration of p53 function leads to tumour regression in vivo. Nature, 445(7128), 661-665. doi:10.1038/nature05541
