# Supplementary figures and images for: Targeting RAS‐converting enzyme 1 overcomes senescence and improves progeria‐like phenotypes of ZMPSTE24 deficiency
Source: Aging Cell. 2020 Jul 24;19(8):e13200. doi: 10.1111/acel.13200 (PMC7431821; doi:10.1111/acel.13200)

Supplemental Figure 1

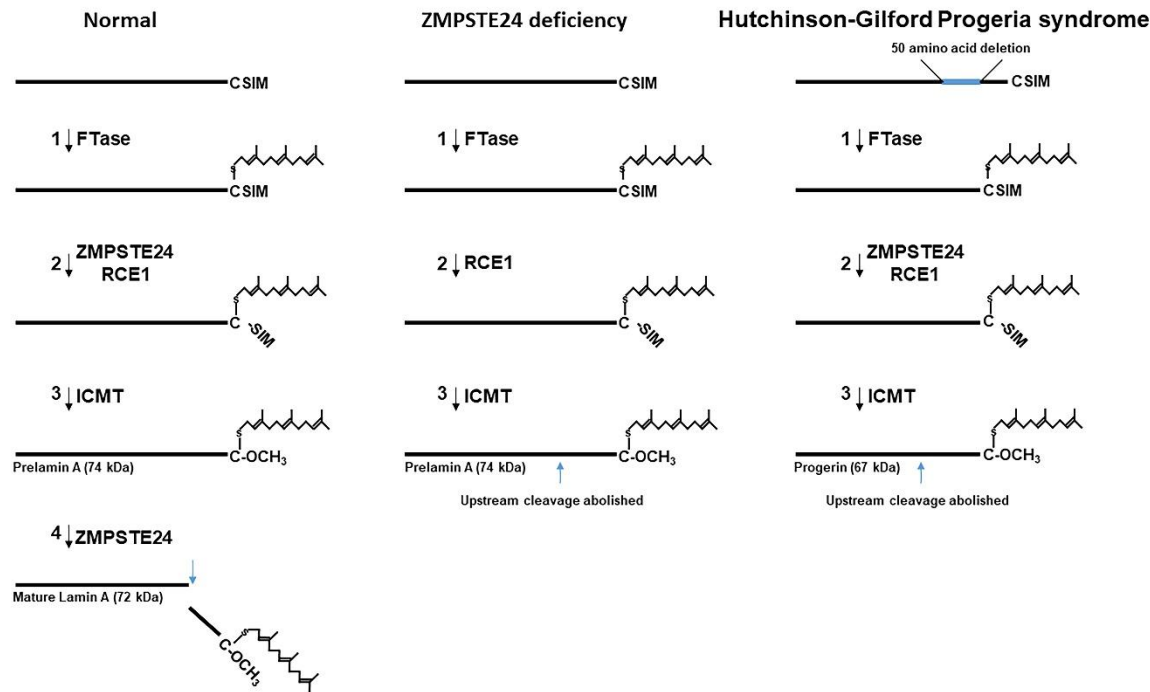

## Supplemental Figure 2

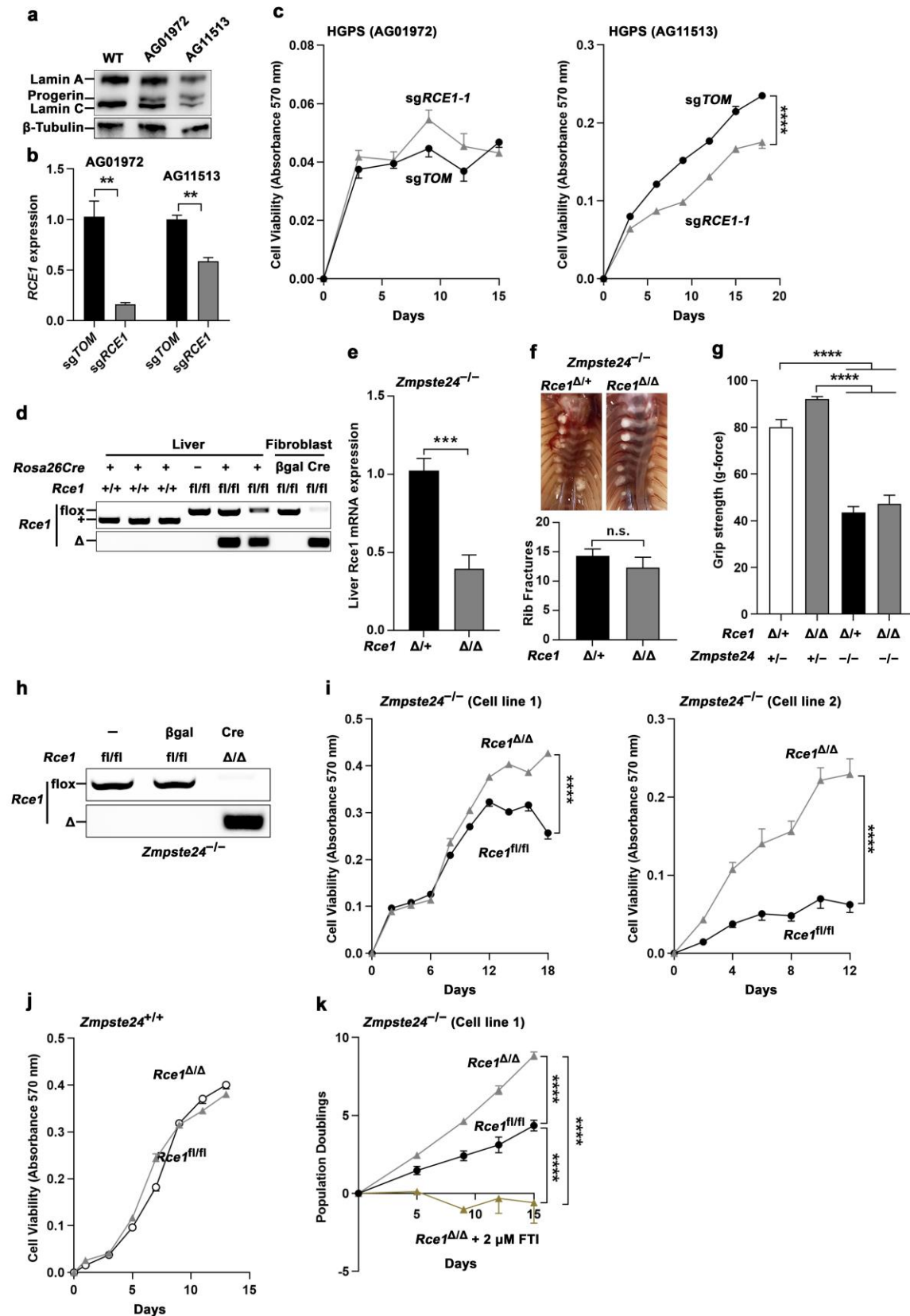

Supplemental Figure 3

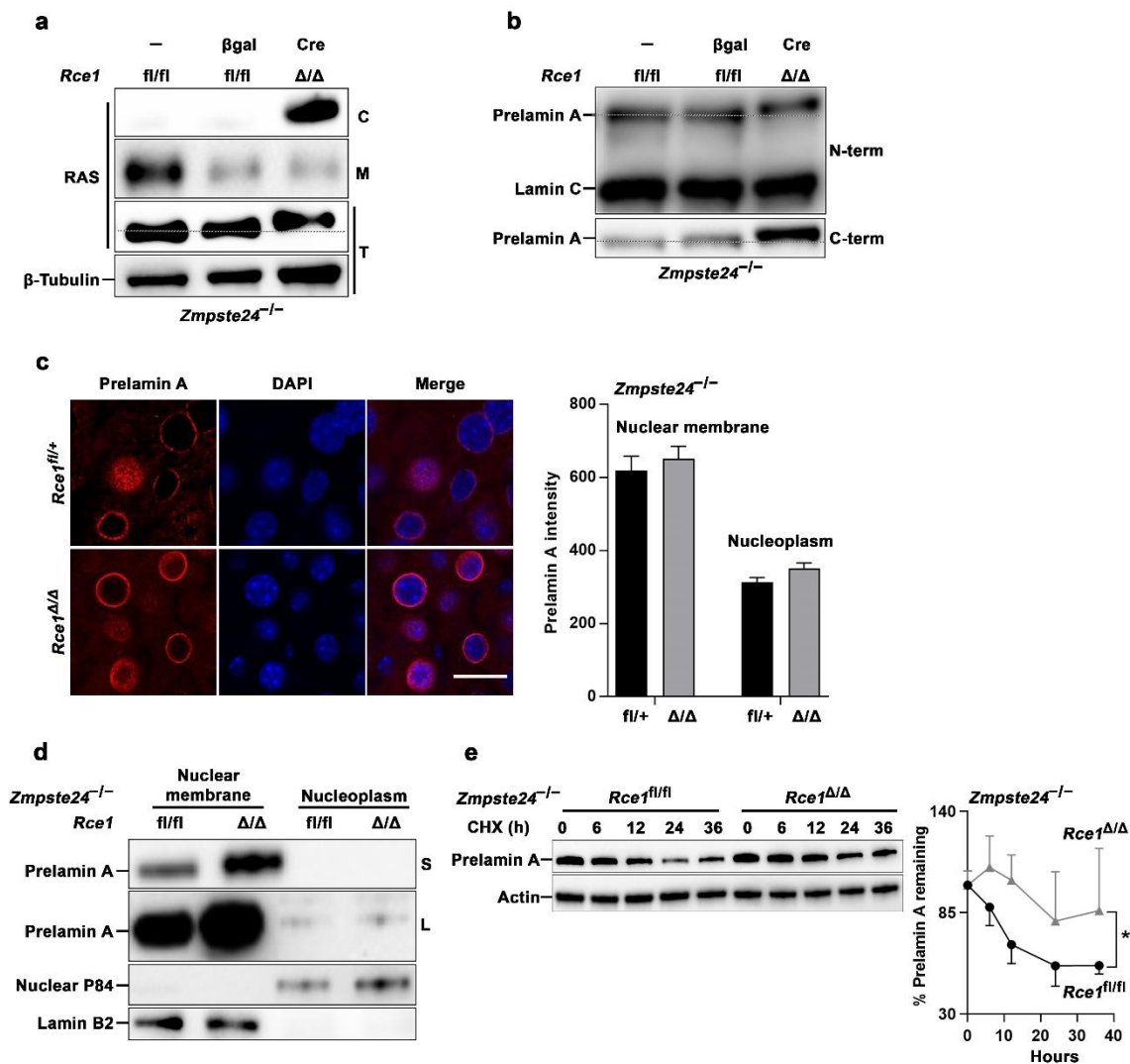

Supplement: Supplementary file 2 — Supplementary Material [file ACEL-19-e13200-s002.pdf]
